# High intratumoral metastasis-associated in colon cancer-1 expression predicts poor outcomes of cryoablation therapy for advanced hepatocellular carcinoma

**DOI:** 10.1186/1479-5876-11-41

**Published:** 2013-02-15

**Authors:** Yong-Ping Yang, Jian-Hui Qu, Xiu-Juan Chang, Yin-Ying Lu, Wen-Lin Bai, Zheng Dong, Hong Wang, Lin-Jing An, Zhong-Xian Xu, Chun-Ping Wang, Zhen Zeng, Ke-Qin Hu

**Affiliations:** 1 Center of Therapeutic Research for Liver Cancer, the 302nd Hospital, 100 Xi Si Huan Middle Road, Beijing 100039, China; 2 Beijing Institute for Infectious Disease, Beijing, China; 3 Division of Gastroenterology/Hepatology, University of California, 101 the City Dr., Building 56, Ste. 237, Irvine, CA 92868, USA

**Keywords:** Cryoablation, Metastasis-associated in colon cancer-1 (MACC1), Advanced hepatocellular carcinoma, Efficacy, Safety, Outcomes

## Abstract

**Background:**

Cryoablation is one of the local therapies for hepatocellular carcinoma (HCC), but its safety and effect has not been studied in patients with Child class A or B and Barcelona Clinic Liver Cancer (BCLC) stage C HCC. Metastasis-associated in colon cancer-1 (MACC1) overexpression has been associated with poor prognosis of HCC, but its predictive value to post-cryoablation outcomes remains unknown in patients with BCLC stage C HCC.

**Methods:**

This study assessed the safety and outcomes of cryoablation measured by time to progression (TTP) and overall survival (OS), and predictive value of MACC1 mRNA and protein overexpression in tumorous tissue to post-cryoablation outcomes in 120 advanced HCC patients with child-pugh class A or B by quantitative polymerase chain reaction and immunohistochemical staining. The potenial correlation of MACC1 and c-Met expression to tumor cell proliferation and apoptosis was also analyzed.

**Results:**

The cryoablation in patients with advanced unresectable HCC resulted in a median TTP and OS of 5.5 (4.2- 6.7) months and 10.5 (9.0-12.0) months, respectively and no significant complications, comparable to the historical report for RFA therapy. The MACC1 mRNA and nuclear protein expression was significantly increased in tumorous tissues in these patients than that in normal liver tissue controls. Higher expression of MACC1 mRNA and nuclear protein in tumorous tissues in these patients was associated with shorter post cryoablation median TTP and OS than that with lower MACC1 expression.

**Conclusions:**

Cryoablation is a safe and effective therapeutic option for patients with advanced HCC and Child-pugh class A or B cirrhosis; and a higher intratumoral expression of MACC1 or nuclear translocation predicts poor outcomes of cryotherapy in these patients.

## Background

Hepatocellular carcinoma (HCC) is the fifth most common cancer and the third most common cause of cancer-related death worldwide [1]. Patients with advanced HCC carry poor outcomes [2], and treatment has been limited [3,4] until recent application of sorafenib that prolong survival rates in a limited way in these patients [5,6]. Cryoablation has been reported as a valid alternative to surgery for HCC treatment in patients with cirrhosis [7]. The main advantage of percutaneous cryotherapy over surgical resection is that it can be used to selectively destroy HCC tumorous tissue while sparing more non-tumoral liver tissue, and therefore, better reserve liver function [8]. This is particularly important to HCC, because the majority of these patients have cirrhosis and compromised liver function. Recently, Osada et al. and our group found that cryotherapy causes not only local tumor necrosis, but also the adjacent tumor tissue was necrotic and shrunken in HCC patients following cryoablation, which is considered as ectopic tumor suppression [9,10]. Cryotherapy might also improve host’s immunity following the treatment. We have recently reported that the frequency of regulatory T cells had significantly decreased in patients with HCC regression following cryoablation, but dramatically increased in patients with HCC recurrence [11]. These indicated that besides HCC ablation, cryotherapy might also function as a systemic treatment by improved immunity. Although cryoablation has not been widely accepted for treatment of advanced HCC, studies reported this modality could prolong survival in patients with advanced HCC [12]. Although a large volume of literature on cryotherapy results in cryo-immunology response [11,13], there are many unanswered questions regarding the role of cryoablation in HCC treatment. Although cryoablation has been reported to prolong survival in unresectable advanced HCC, the sample size was too small to draw a reliable conclusion [12]. Thus, randomized controlled studies are needed to compare cryoablation with other therapies, such as resection and Radiofrequency Ablation (RFA).

Metastasis-associated in colon cancer-1(MACC1), a new gene associated with primary and metastatic colon cancer, promotes tumor cell growth as well as the development of distant metastasis [14]. Overexpression of MACC1 induces down-stream activation of HGF/c-Met and promotes metastasis of colon cancer, while silencing of MACC1 leads to reduced tumor proliferation, decreased cell migration, and a lack of new metastasis [15,16]. Our recent studies found that overexpression of MACC1 predicts a poor outcome in patients with HCC after hepatectomy [17]. Together with other reports [18], these findings imply that MACC1 might serve as a novel prognostic marker in HCC. Thus, we assessed whether intratumoral MACC1 is overexpressed and associated with clinical outcomes in advanced HCC after cryoablation.

In the present study, we prospectively followed patients who had undergone cryoablation for unresectable Barcelona Clinic Liver Cancer (BCLC) stage C HBV-related HCC in a relatively large clinical cohort of patients than earlier studies. The main purpose of this study was to evaluate the safety and effects of percutaneous cryoablation for advanced HCC. We also assessed the distribution of MACC1 expression and value of predicting the post cryoablation outcomes in these patients.

## Methods

### Study concept

The study protocol was approved by the 302nd Hospital Research Ethics Committee, and written informed consent was obtained from all participants. The patients undergoing cryoablation therapy and qualified the inclusion criteria below at two tertiary medical centers from February 2008 and August 2010 were prospectively followed. The primary endpoint of the study was to determine the time to progression (TTP) after cryoablation. The secondary endpoints included overall survival (OS), the disease-control rate (DCR) and safety of cryoablation. The association of MACC1 mRNA and protein expression with post-cryoablation OS and TTP was also assessed in the same cohort of patients.

### Patients

A total of 120 patients with advanced HBV-related HCC who underwent cryoablation therapy and qualified the following inclusion criteria were selected from 378 consecutive patients and followed in Beijing the 302^nd^ Hospital, China. The inclusion criteria were: (A) diagnosis of HCC by histologic confirmation and stage of unresectable BCLC staging C with portal vein thrombosis(PVT), as a sign of macroscopic vascular invasion [4]; (B) at least one tumor lesion that could be measured along one dimension according to the Response Evaluation Criteria in Solid Tumors (RECIST) v1.1 criteria [19]; (C) presence of portal hypertension and Child class A or B cirrhosis by endoscopy, imaging, and clinical presentation with life expectancy of at least 12 weeks [20]; (D) Eastern Cooperative Oncology Group Performance Status (ECOG PS) of 0, 1 or 2; (E) total bilirubin ≤ 51.3 μmol/L; (F) HBsAg positive and anti-HCV-negative; and (G) regular and minimal 3 months of follow-up prior to and post cryoablation. None of the patients had received any prior HCC treatment. Histological grade of HCC differentiation was classified by Edmondson criteria into well, intermediate, and poorly differentiated [21].

A total of 258 patients were excluded, including 51 patients were Child-Pugh C; 44 patients with Child-Pugh B, but serum bilirubin level >51.3 μmol/L; 25 patients with life expectancy < 12 weeks; 13 patients with ECOG PS ≥ 3 and 69 patients with a history of prior HCC treatment (hepatectomy = 9, preoperative chemotherapy = 7, prior TACE or local ablation = 45, radiotherapy = 8); 36 patients extrahepatic spread; and 20 patients missed follow-up in 3 months after cryoablation.

HCC tumorous samples were obtained from all enrolled 120 patients at the time of cryoablation, and prepared for histochemical staining or snap-frozen in liquid nitrogen for RNA extraction and reverse transcription-polymerase chain reaction (RT-PCR). Microdissection was not performed in current study. Part of the tissue specimens were fixed in 10% formalin and paraffin-embedded for hematoxylin and eosin (H&E) and immunohistochemical staining (IHS). Because nontumoral tissue was not routinely collected during diagnostic biopsy, ten normal liver tissues were obtained as controls, including 4 from cases with hepatic hemangioma and 6 with hepatic cyst. None of these had history of viral hepatitis or cirrhosis.

### Cryoablation procedure

Cryoablation was performed as previously described [22]. Briefly, the cryoablation procedure was performed under conscious sedation. Echocardiography, ventilation and oxygen saturation levels were monitored throughout the procedure. Patients were kept warm during cryoablation with warming mats.

Argon-helium gas-based CRYO care system (EndoCare, Inc., CA, USA) and cryoprobes (2 or 3 mm) were used to freeze the tumor with a dual freeze-thaw cycle under ultrasound or computerized tomography (CT)-guidance. The cryoprobe temperatures were reduced to −135 ± 2°C within 1 min. The dual freeze-thaw cycle comprised a 20-min freeze, a 10-min thaw and a further 15-min freeze. After removal of the probes, all tracts were packed with Surgicel (Johnson & Johnson, Inc., Arlington, TX, USA) through the sheath introducer to control bleeding, and the sheath introducer was removed. The size and number of cryoprobes were determined depending on the location and the average size of the HCC lesions to be ablated. To maximally ablate tumor lesions, cryoablation was performed once or up to a total of 3 sessions as needed. To our experience, tumors less than 5 cm in diameter can be completely ablated and tumors larger than 5 cm can be reduced by at least 70%.

### Assessment of the clinical outcomes

#### Cryoablation treatment response

This was assessed by CT scan or magnetic resonance imaging (MRI) approximately every 8 weeks via independent radiologists and classified according to RECIST v.1.1 assessment [19], i.e., complete response (CR), partial response (PR), stable disease (SD), or progressive disease (PD). Patients who achieved CR, PR or SD were defined as achieving clinical benefits (CB). Patients who showed CR or PR were defined as achieving a clinical efficacy response (CER), whereas disease control rate (DCR) is defined as the total rate of CR, PR and SD.

#### Assessment of OS and TTP after cryoablation treatment

The OS was defined as the time from cryoablation initiation to the date of death or the patient’s last follow-up [23]. TTP was defined as the time from cryoablation initiation to the date of disease progression or death. The disease progression was defined as the tumor progression according to RECIST v.1.1 or progression of cirrhosis.

#### Safety assessment of cryoablation treatment

After the treatment, all patients were observed in an intensive care unit overnight to monitor for life-threatening delayed hemorrhage and other complications, such as cryoshock syndrome (chills, fever, tachycardia, tachypnea and temporary renal damage), liver abscess, intestinal fistulas, fever, myoglobinuria, pulmonary edema, pneumothorax, thrombocytopenia, pain, and skin frostbite. Follow up postoperative management [24].

### MACC1 mRNA expression with post-cryoablation OS and TTP

#### Quantitation of MACC1 mRNA by RT PCR

The MACC1 mRNAs were quantified by standard reverse transcription polymerase chain reaction (RT-PCR) [14,17] assay blindly to the clinical records. The following PCR primers were used for MACC1 cDNA (136 bp), 5^′^-TTCTTTTGATTCCTCCGGTGA-3^′^(F) and 5’-ACTCTGATGGGCATGTGCTG-3’(R). Briely, total RNA was isolated from the tissues by using an RNA isolation kit (Qiagene, Germany). The extracted RNA was quantified by spectrophotometric measurement at A260, and the purity was verified by the A260/A280 ratio >1.8. A total of 2 μg RNA was used for the production of cDNA by reverse transcriptase-PCR (SYBR PrimeScript RT-PCR Kit with SYBR Premix Ex Taq; Takara, Japan). The cDNA templates were subjected to PCR amplification under the following conditions: pre-denaturation at 95° for 30 sec; 28 cycles of denaturation at 95° for 5 s, annealing at 60° for 30 s and extension at 72° for 30 s; and final extension at 72° for 10 min. The final products were analysed by 2.0% agarose gel electrophoresis and stained with ethidium bromide. Each PCR was performed in triplicates. The threshold cycle (Ct) value for triplicate reactions was averaged, and the relative genomic expression was calculated by 2^-ΔCt^ value [ΔCt = Ct (MACC1) - Ct (β-actin)] [25].

To assure the assay was not contaminated by trace amount of DNA, PCR reactions were also performed in RNA control samples that lacked reverse transcriptase during cDNA synthesis. Each PCR was performed in triplicate. β-actin mRNA (125 bp) measurement was used as an internal control with the following primers: 5^′^-CGGGAAATCGTGCGTGAC-3^′^ (F) and 5^′^-AGGCAGCTCGTAGCTCTTCT-3^′^ (R).

#### Assessment of MACC1 in HCC tumorous and normal control liver tissue by IHS

Paraffin-embedded HCC tumorous and additional 10 normal control liver tissue from patients with history of hepatic hemangioma (n = 4) or hepatic cyst (n = 6) were used for MACC1 indirect immunohistochemical staining. Polyclonal rabbit anti-human antibody against MACC1 (1:50; Sigma,St. Louis, USA) was used as primary antibody, peroxidase-conjugated anti-mouse/rabbit immuoglobulin (polymer Detection system, GBI, Mukilteo, USA) as the secondary antibody, followed by 3-amino-9-ethylcarbozole (AEC; GBI, Mukilteo, USA). For the negative control, PBS was used to replace the first antibody that resulted in a negative staining.

The MACC1 staining results were evaluated and characterized by two independent pathologists. The amount of positive MACC1 staining, shown in red, was scored as previously reported [26]: 0, ≤10%; 1, >10–25%; 2, >25–50%; 3, >50–75%; and 4, >75%. The intensity of the special staining was scored as follows: 0, negative; 1+, weak; 2+, moderate; and 3+, strong. The final score was obtained by multiplying the extent scores and intensity scores, which produced values in a range from 0 to 12. A score of 0–4 was defined as a negative MACC1 expression (−); a score of 6–8, an intermediate positive MACC1 (+); a score of 9–12, a strong positive MACC1 (++).

### Cell lines

Human hepatoma cell lines (HepG2, MHCC-97H, MHCC-97 L) and the immortal nontumourigenic normal human hepatocyte cell line(L-02) were used to screen the expression of MACC1 and c-Met. HepG2 was purchased from the American Type Culture Collection (Manassas, VA). L-02, MHCC-97H and MHCC-97 L were obtained from the Type Culture Collection of the Chinese Academy of Sciences (Shanghai, China) and maintained under recommended culture conditions. Cells were grown at 37°C in a humidified incubator containing 5% CO2 and spread onto 6-well chamber slides for mRNA, western blot analysis and cell transfection. PCR primers for c-Met mRNA detection were also as described in our previous report [17]. Protein extraction, immunoblot analysis for MACC1 and c-Met were performed by Western blotting as descibed [13]. Antibody against c-Met(diluted 1:1000) and MACC1(diluted 1:800) were used as primary antibody. β-actin-specific antibody served as loading controls(diluted 1:2000; sigma Chemical Co., USA ).

### SiRNA Transfection, apoptosis and cell cycle analysis

Two pairs of sequences of small interfering RNA (siRNA) targeting MACC1 called MACC1-siRNA1, MACC1-siRNA2 and negative control (si-Control) were designed and synthesized by Guangzhou Borui Bioengineering (Guangzhou, China) and their sequences are as follows: MACC1-siRNA1: 5^′^-CAA GGA AGU UUC UGU AUG ATT-3^′^, MACC1-siRNA2: 5^′^-GAA AUA ACA GGA AGA GAA ATT-3^′^. Transfection of siRNA into MHCC-97H and MHCC-97 L was carried out using Lipofectamine 2000 (Invitrogen, Carlsbad, CA) and Opti-MEM (Invitrogen, Carlsbad, CA), according to the manufacturer’s protocol. Transfection efficiency was monitored by pEGFP-N1 plasmid (clontech), with 60%-70% efficiency. Cells were harvested 48 h after siRNA transfection for mRNA, protein, apoptosis and cell cycle analysis. Cells were stained with Annexin V using cell Apoptosis Detection Kit for cell apoptosis analysis, or treated with PI staining solution (0.03% Triton X-100, 5 mg/ml Rnase A and 10 ug/ml propidium iodide) to determine cell cycle status on a flow cytometer. The results are representative of three independent experiments with triplicate samples for each condition.

### Statistical analysis

All statistical analysis was performed with SPSS version 16.0 software. Continuous data were expressed as median and range. A comparison between the groups was performed using the χ2 test. Survival rates were estimated by the Kaplan-Meier method and compared by the log rank test. The Cox proportional hazards model was used to determine the independent factors on survival, based on the variables selected in univariate analysis. *P* < 0.05 was considered statistically significant.

## Results

### Patient characteristics and clinical outcomes

**Figure 1:**
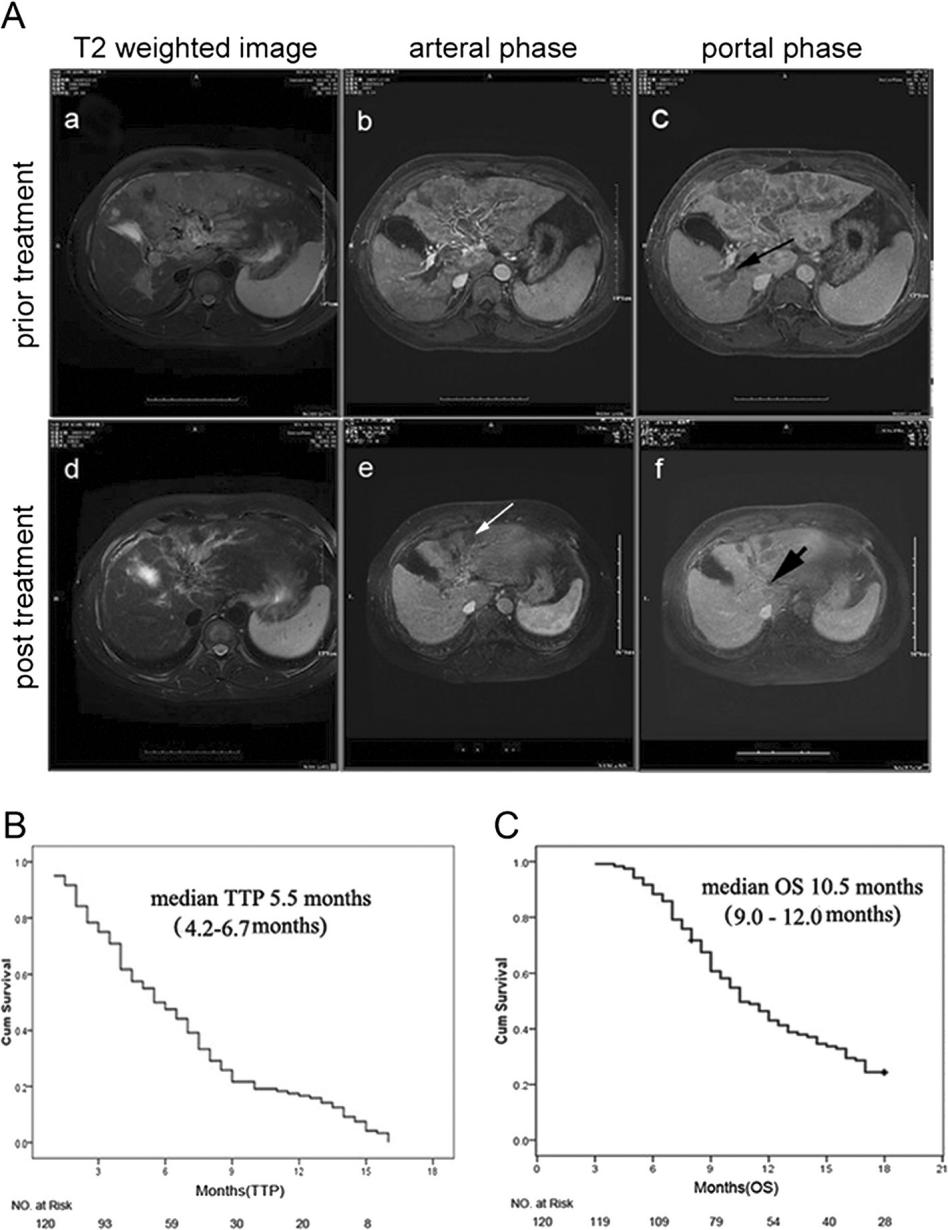
**Efficacy of cryoablation therapy for advanced hepatocellular carcinoma. A**: a complete response in a 50-year-old man survived for over 24 months after cryoablation. MRI scan showed a huge mass (M) at the dome of the left hepatic lobe (**a**: T2 weighted image; **b**: arterial phase). Long black arrow(b)indicated portal vein thrombosis (**c**: portal phase) with histopathological confirmation of HCC embolus, and Edmondson classification of intermediate differentiation. Ten months after two cryoablation therapies, 60% reduction was achieved from the baseline tumor burden, also associated with the decrease of the non-treated adjacent tumor (**d**). Long white arrow indicated the treated tumor was necrotic (**e**). Short black arrow indicated portal vein tumor thrombus was almost invisible after cryoablation (**f**). **B**: Kaplan-Meier survival curves are shown for 120 advanced patients treated with cryotherapy and the median TTP for the whole cohort was 5.5 months (95% CI 4.2 –6.7 months). **C**: Kaplan-Meier estimates of median overall survival for advanced patients treated with cryotherapy was 10.5 months (95% CI 9.0–12.0 months).

Baseline characteristics are presented in Table 1. A total of 285 cryoablations were performed in 193 tumors in 120 patients with unresectable HBV-related advanced **HCC**, with 1, 2 and 3 procedures in 17, 41 and 62 patients, respectively. The median follow-up was 9 months (range 3–18 months) and the median OS and TTP for the whole cohort were 10.5 months (95% confidence interval [CI] 9.0–12.0 months) and 5.5 months (95% CI: 4.3 –6.7 months), respectively (Figure 1). Regarding the analysis for best response, 5 of 120 (4.2%) patients in cryotherapy exhibited CR (Figure 1A), 15 (12.5%) patients achieved PR, and 55 (45.8%) patients achieved SD lasting >8 weeks. Furthermore, 92 (76.7%) patients who died, 28 (30.4%) were due to esophagogastric varices bleeding; 16 (17.4%), refractory ascites-induced renal failure; 14 (15.2%), liver failure; 24 (26.1%), recurrence/metastasis; and 10 (10.8%), tumor rupture/hemorrhage. Overall, 58/92 (63%) died of cirrhosis related complication; and 34/92 (37%) died of HCC related complications.

**Table 1:** Baseline clinical data of 120 patients with advanced HCC

**Presentation**	**Cases (%)**
Gender	
male	106(88.3%)
Age(yrs), median (range)	48 (28–72)
ECOG PS	
0	25(20.8%)
1	55(45.8%)
2	40(33.3%)
HCC differentiation	
well	20(16.7%)
moderately	50(41.7%)
poorly	50(41.7%)
HCC diameter (cm),median (range)	6 (2.4-12.6)
Tumor number	
1	67(55.8%)
2	33(27.5%)
3	20(16.7%)
Invasion to portal vein	
Branch	71(59.2%)
Trunk	49(40.8%)
HBV DNA	
Positive	76(63.3%)
Child-Pugh class	
A	58(48.3%)
B	62(51.7%)
AFP(ng/ml), median (range)	1368 (7–20000)

*ECOG*: Eastern Cooperative Oncology Group;

*HBV*: hepatitis B virus; *AFP*: alfa-fetoprotein.

### Safety assessment

#### Cryoablation procedure-related adverse events

Major complications occurred in 8 (6.7%) patients. Four (3.3%) cases developed hepatorrhexis bleeding into the peritoneal cavity diagnosed 3–6 hours after the cryoablation procedure, and all were successfully managed by immediate transcatheter arterial embolization (TAE) and supportive measures. Cryoshock syndrome occurred in 3 (2.5%) cases with larger tumor load (i.e., a mean total estimated area of HCC > 50–60 cm^2^). All these 3 patients recovered following intravenous atropine and 5% sodium bicarbonate, covering with an electric blanket, and oxygen inhalation. One patient developed a liver abscess at the ablation zone that was not absorbed, and the abscess content exuded from a scar of the probe tract 1 month after cryoablation, and required drainage.

Other post cryoablation complications included fever (52/120, 43.3%), pain (47/120, 39.2%), skin frostbite (41/120, 34.2%), right pleural effusion (39/120,32.5%), thrombocytopenia (31/120, 25.8% included 2 patients with platelet count decreased more than 50% from pretreatment, 29 patients with mildly decreased platelet from baseline, which returned to the baseline for about 1 week after cryoablation), pneumothorax (4/120, 3.3%), myoglobinuria (3/120, 2.5%), and 2 patients (1.6%) developed stress-related gastric mucosal lesion-induced hemorrhage and they were treated with acid-inhibiting and gastric mucosa-protecting agents.

#### Worsening liver function status

Percutaneous cryoablation caused transient and mild elevation in alanine aminotransferase (< 5 × ULN, 94/120, 78.3%) and total bilirubin (< 2 × ULN, 21/120, 17.5%) levels. All these returned to baseline levels about 2 weeks after cryoablation therapy (P > 0.05). However, hepatic decompensation or liver failure occurred in 5 (4.2%) patients with Child-Pugh class B (score = 8) and trunk PVT, including 3 developed new onset or worsening ascites, and 2 developed liver failure. Three of these 5 patients recovered to baseline and two died of liver failure.

### Association of intratumoral MACC1 expression with clinical presentation and cryoablation therapy-related outcomes

#### MACC1 mRNA expression

**Figure 2:**
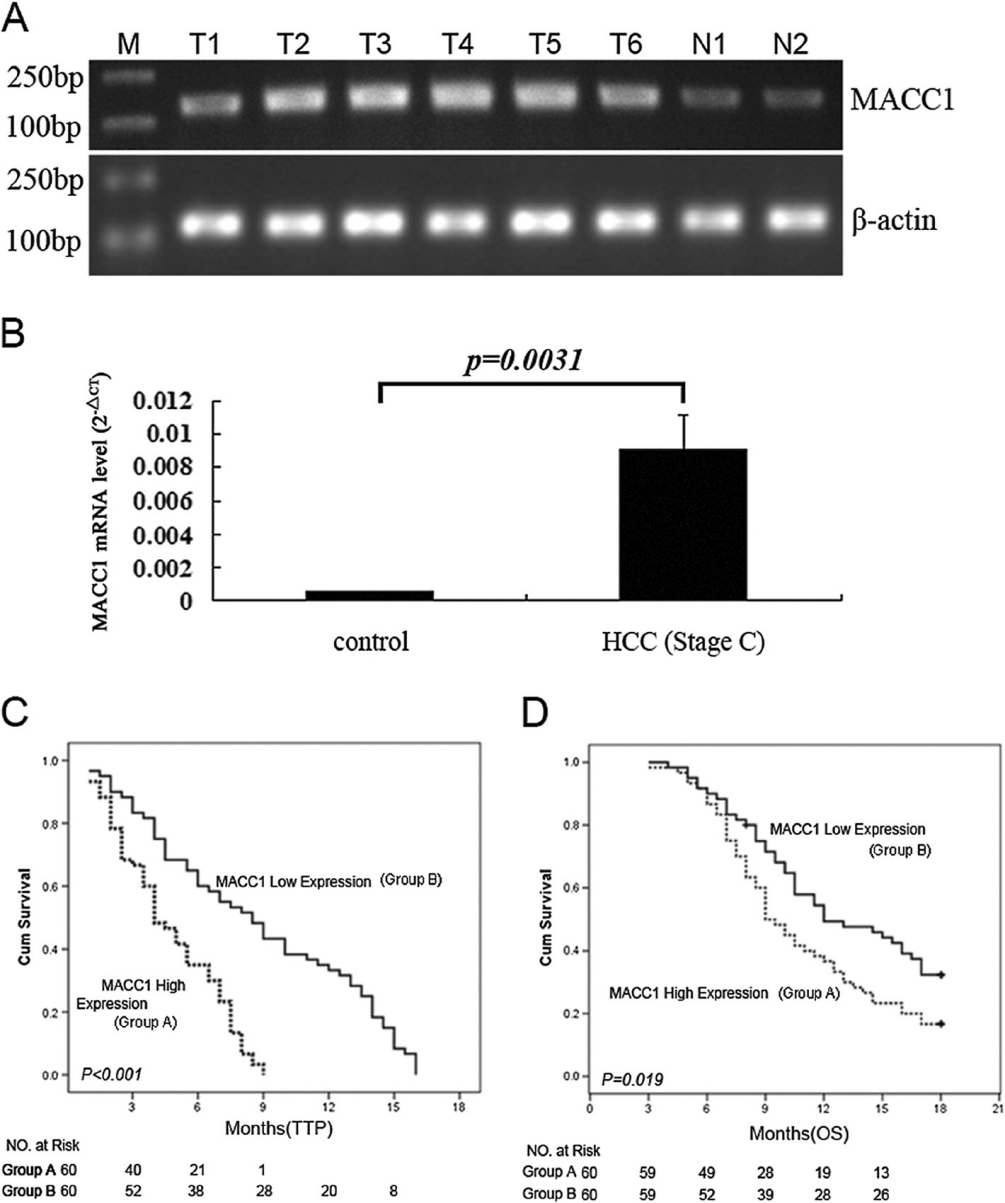
**Expression of MACC1 and the outcomes of advanced HCC after receiving cryoablation therapy. A**: MACC1 mRNA expression measured by RT-PCR in tumorous tissue from patients with advanced HCC (upper panel, T1-T6) vs. normal liver tissue from controls (upper panel, N1 and N2), and the lower panel was β-actin as internal controls. **B**: Mean MACC1 mRNA expression in tumorous tissue from patients with advanced HCC (right) vs. that in control (left) by real-time RT-PCR. **C**: Kaplan–Meier estimates of time to progression in patients with higher MACC1 mRNA expression vs. those with lower MACC1 mRNA expression (p < 0.001). **D**: Kaplan–Meier estimates of overall survival time in patients with higher MACC1 mRNA expression vs. those with lower MACC1 mRNA expression (p < 0.019).

By quantitative RT-PCR assay, the level of intratumoral MACC1 mRNA expression in these advanced HCC patients (0.009 ± 0.005) was about 15-fold higher than that in 10 normal control liver tissue (0.0006 ± 0.00005, p = 0.0031, Figure 2A and 2B). According to the median value of intratumoral MACC1 mRNA expression (0.009, range: 0.0051–0.0139), these 120 cases were divided into 2 groups: those with low intratumoral MACC1 expression (<0.009) and those with high intratumoral MACC1 expression (≥0.009). A higher expression of MACC1 mRNA was significantly associated with ages, tumor size, portal vein trunk invasion, multinodular and poorly tumor differentiation, but not with gender, lesion number, AFP level or Child-Pugh class (Table 2).

**Table 2:** The associaition of MACC1 mRNA expression with the baseline clinical presentation in advanced HCC patients

**Variables**	**All cases (*n = 120 *)**	**MACC1 mRNA high expression (total cases = 60) [n(%)]**	**MACC1 mRNA low expression (total cases = 60) [n(%)]**	**P value**
**Gender**				
Male	106	52(87)	54(90)	0.7761
**Age**				
≥ 48 years	60	25(42)	38(63)	0.0283
**Tumor size**				
≥ 6 cm	60	37(62)	18(30)	0.0010
HCC thrombus				
Portal trunk	49	32(53)	17(28)	0.0093
**HCC number**				
Multinodular	53	32(53)	21(35)	0.0432
**Differentiation**				
Well	20	8(13)	12(20)	0.0347
Moderately	50	20(34)	30(50)	
Poorly	50	32(53)	18(30)	
**Serum AFP**				
≥1368	85	41(68)	44(73)	0.6879
**HBV DNA**				
Positive	76	36(60)	40(67)	0.4486
**Child-Pugh**				
A	58	31(52)	27(45)	0.4650
B	62	29(48)	33(55)	

As shown in Table 3, the overall CER and DCR in this cohort of patients with advanced HCC and received cryoablation were 16.7% (20/120) and 62.5% (75/120), respectively. Compared to those with low intratumoral MACC1 mRNA expression, patients with high intratumoral MACC1 expression had a low CER and DCR (8.3% vs. 25.0%, p = 0.0275 for CER; 51.7% vs. 73.3%, p = 0.0273 for DCR). Moreover, the advanced HCC patients with MACC1 nuclear staining had a poorly CER and DCR than those with MACC1 cytoplasmic staining (12.8% vs. 16.7%, p = 0.843 for CER; 43.6% vs. 68.8%, p = 0.0321 for DCR).

**Table 3:** MACC1 expression and outcomes of post-cryoablation therapy

	**MACC1 mRNA expression**		**P**	**MACC1 protein localization**		**P**
	**High (n = 60)**	**Low (n = 60)**		**Cytoplasmic (n = 48)**	**Nuclear (n = 39)**	
TTP	4.0	8.5	< 0.001	5.0	4.0	<0.001
	3.0-5.0(M)	6.3-10.7 (M)		4.2-5.8 (M)	3.6-4.4 (M)	
OS	9.0	12.0	0.019	11.0	8.0	0.001
	7.7-10.3 (M)	7.8-16.2 (M)		8.1-13.9(M)	6.7-9.2(M)	
Overall response rate (CR + PR)	5(8.3%)	15(25.0%)	0.027	8(16.7%)	5(12.8%)	0.843
Disease control rate (CR + PR + SD)	31(51.7%)	44(73.3%)	0.027	33(68.8%)	17(43.6%)	0.0321

Abbreviations: Overall response rate: includes complete and partial response. Disease control rate: includes complete, partial response and stable disease. *M*: months.

The median post cryoablation TTP was 4.0 (95% CI: 3.0- 5.0) months in the group with higher intratumoral MACC1 mRNA expression, that was significantly lower than the median TTP of 8.5 (95% CI: 6.3- 10.7) months in the group with lower intratumoral MACC1 mRNA expression (log-rank P < 0.001, Figure 2C). In addition, a significantly shorter median post cryoablation OS (9.0 months; 95% CI: 7.7- 10.3 months) was seen in the group with higher intratumoral MACC1 mRNA expression than that (12.0 months; 95% CI: 7.8- 16.2 months) in the group with lower intratumoral MACC1 mRNA expression (log-rank P = 0.019; Figure 2D). To further determine the association of MACC1 mRNA expression with post cryoablation outcomes, the Cox hazard model was used for multivariable analysis. As shown in Table 4, the multivariate analysis confirmed that OS after cryoablation treatment for treating advanced HCC were independently associated with ECOG PS and overexpression of intratumoral MACC1 mRNA,and then TTP associated with high expression of intratumoral MACC1 mRNA.

**Table 4:** Multivariate analysis of prognosis factors associated with post-cryoablation survival in 120 patients with advanced HCC

**Variables**	**Multivariate**		
	**RR**	**CI 95%**	**P**
**Time to progression**			
Age	0.465	0.201-1.128	0.082
ECOG PS	1.428	1.014-1.823	0.221
Child-Pugh class	1.126	1.051-1.517	0.329
Tumor diameter	0.721	0.437-1.207	0.221
Tumor number	0.938	0.783-1.178	0.746
Tumor differentiation	1.246	0.752-1.972	0.384
MACC1 mRNA expression	2.103	1.378-2.801	0.005
HCC thrombus in PV	1.033	0.662-1.734	0.881
**Overall survival**			
Age	0.569	0.301-1.216	0.278
ECOG PS	1.452	1.021-2.033	0.026
Child-Pugh class	1.127	1.038-1.861	0.105
Tumor diameter	1.221	0.332-1.325	0.763
Tumor number	0.873	0.661-1.148	0.332
Tumor differentiation	1.142	0.673-2.116	0.736
MACC1	1.842	1.201-2.514	0.011
HCC thrombus in PV	1.058	0.483-2.354	0.825

*PV*: portal vein.

#### MACC1 Protein expression

**Figure 3:**
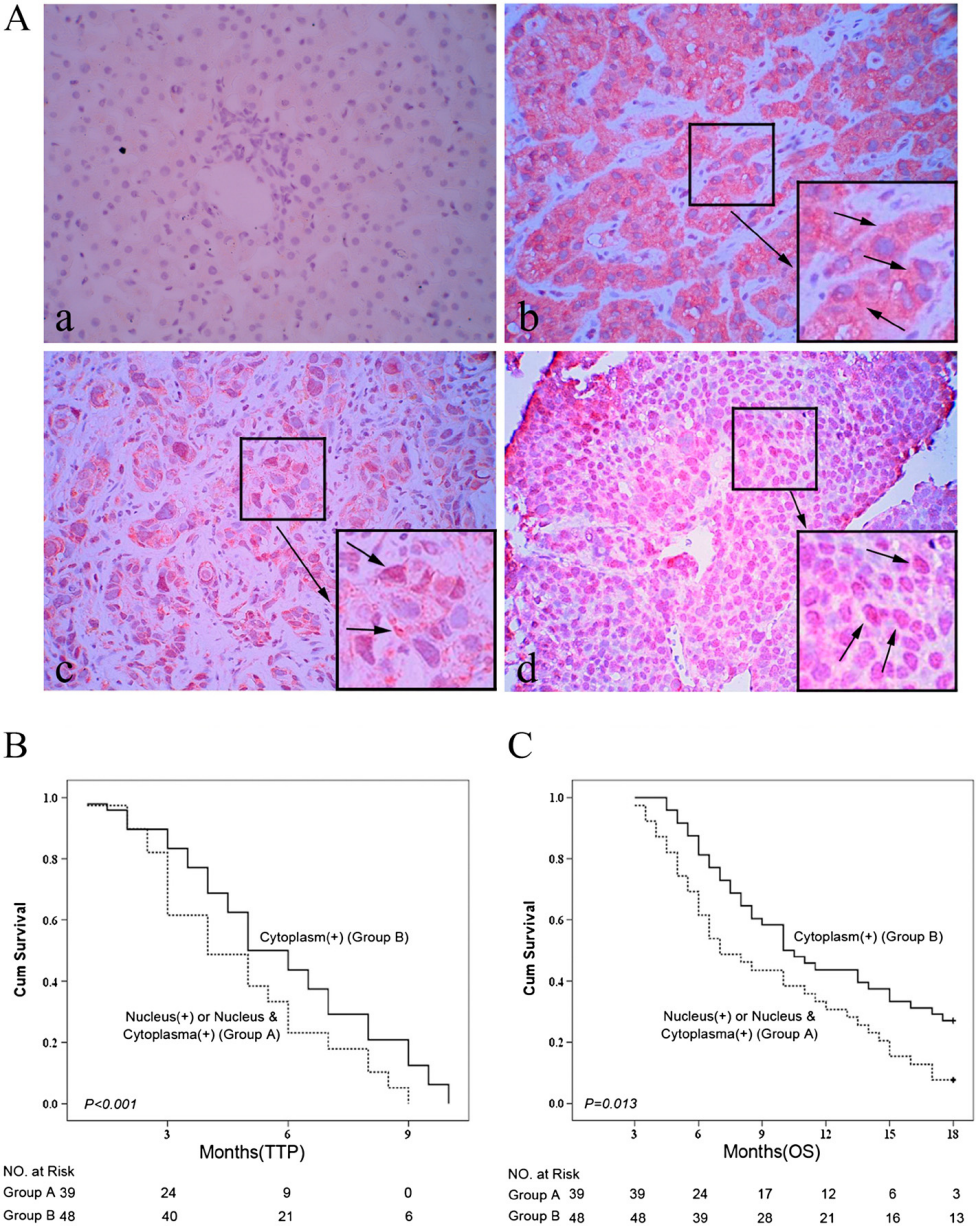
**Nuclear expression of MACC1 protein and overall survival after cryoablation therapy. A**. The expression of MACC1 protein detected by immunohistochemistry (×400). **a**. Normal control liver tissue showed a weak MACC1expression in cytoplasm. **b**. A HCC case showed a mainly cytoplasmic MACC1 expression in tumor tissue (arrowhead). **c**. Another case with both weak cytoplasmic and sporadic nuclear MACC1 expression(arrowhead). **d**. The predominant nulear MACC1 expression in the tumorous tissue of a HCC patient (arrowhead). **B**. Kaplan–Meier estimates of time to progression in patients with nuclear MACC1 expression vs. those with cytoplasmic MACC1 expression (p < 0.001). **C**: Kaplan–Meier estimates of overall survival time in patients with nuclear MACC1 expression vs. those with cytoplasmic MACC1 expression (p < 0.01).

We then determined expression and distribution of MACC1 protein by IHS in the same HCC tumorous tissue versus normal liver tissue controls. Absent or weak cytoplasmic MACC1 expression in the normal control livers tissue was observed (Figure 3A-a). In contrast, the HCC tumorous tissues revealed variable degrees of cytoplasm and/or nuclei MACC1 staining (nuclear MACC1 positive was defined as at least 10% of nuclei stained). Overall, 87/120 (72.5%) HCC specimens showed positive staining for MACC1, including 39 (44.8%) with nuclear staining and 48 (55.2%) with predominant diffuse cytoplasmic staining (Figure 3A). We found MACC1 nuclear expression were associated with poor HCC differentiation (p = 0.0185) and larger tumor size (p = 0.0175). Compared to patients with MACC1 cytoplasmic staining, those with MACC1 nuclear staining had significantly shorter mean post cryoablation TTP [4.0(3.6- 4.4) months vs. 5.0 (4.2-5.8) months, log-rank: P < 0.001] and OS: [8.0 (6.7- 9.2) months vs. 11.0 (8.1- 13.9) months, log-rank: P = 0.01].

#### MACC1 and c-Met expression associated with tumor cell proliferation and apoptosis

**Figure 4:**
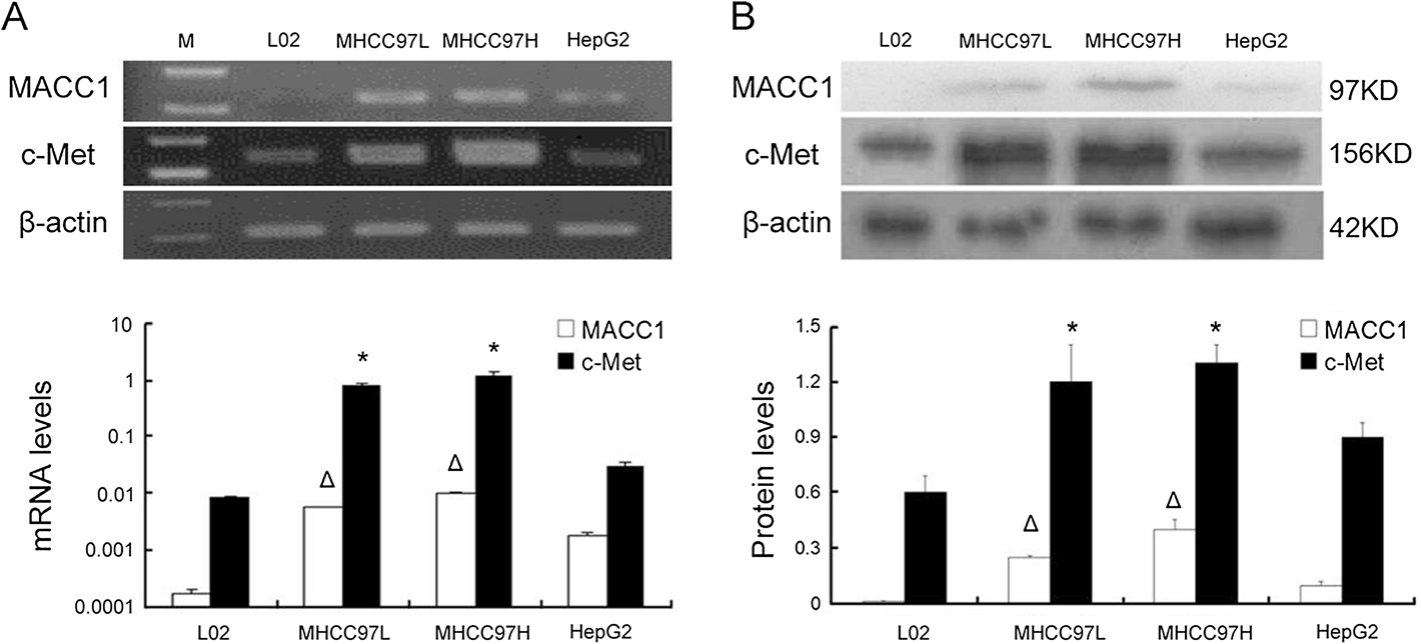
**The expression of MACC1 and c-Met mRNA and protein in liver cell lines. A**: MACC1 and c-Met mRNA in three human hepatoma cell lines (MHCC-97 L, MHCC-97H and HepG2) and one normal liver cell line, L-02, by real-time RT-PCR. (Δp < 0.05 versus HepG2, *p < 0.05 versus L-02 or HepG2). **B**: MACC1 and c-Met protein expression in above cell lines, by western blot. (Δp < 0.05 versus HepG2, *p < 0.05 versus L-02 or HepG2).

**Figure 5:**
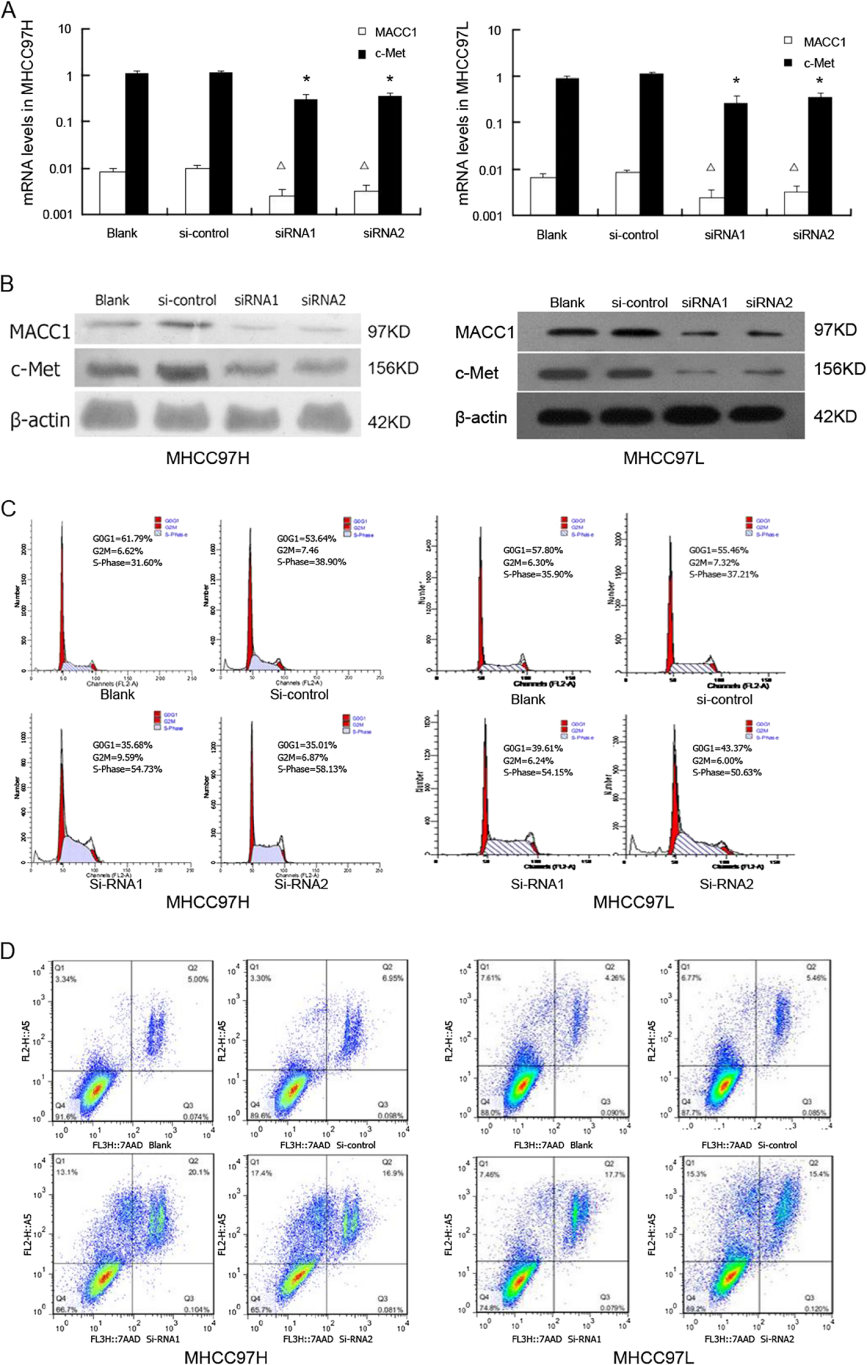
**MACC1 and c-Met expression associated with tumor cell cycle and apoptosis. A**. Comparison of MACC1 and c-Met mRNA levels in MHCC-97H and MHCC-97 L cell lines transfected with two pairs of MACC1-si RNA and control-si RNA, as determined by real-time RT-PCR (Δp < 0.05 versus blank or si-control, *p < 0.05 versus blank or si-control). **B**. MACC1 and c-Met protein expression in MHCC-97H and MHCC-97 L cell lines transfected with two pairs of MACC1-si RNA and control-si RNA, as determined by Western Blot. **C**: The cell cycle analysis in MHCC-97H and MHCC-97 L cell lines transfected with MACC1-siRNA by Flow cytometric analysis. **D**: The percentage of apoptotic cells in MHCC-97H and MHCC-97 L cell lines transfected with MACC1-si RNA by Flow cytometric analysis.

We then examined MACC1 mRNA and protein expression in four liver cell lines by real-time quantitative PCR and western blot. Data showed that there was a lack of MACC1 expression in the normal liver cell line L-02, while all of three hepatoma cell lines (HepG2, MHCC-97L and MHCC-97H) expressed MACC1. MHCC-97L and MHCC-97H showed higher MACC1 transcript levels relative to the HepG2(p < 0.05, Figure 4A). Likewise, MACC1 protein expression was elevated in MHCC-97L and MHCC-97H compared to the HepG2 (p < 0.05, Figure 4B). As for c-Met, both of MHCC-97H and MHCC-97L had higher level than that of L-02 or HepG2 cells (p < 0.05, Figure 4A). After transfection of siRNA targeting MACC1 to MHCC-97H and MHCC-97L cells, which express high level of endogenous MACC1, the MACC1 and c-Met mRNA levels and protein expression were significantly inhibited compared to the control groups (P < 0.05, Figure 5A, 5B). We further investigated if down-regulation of MACC1 expression was associated with tumor cell apoptosis and cycle by using flow cytometric analysis. Cell cycle analysis revealed that MACC1 silencing in MHCC 97H and MHCC 97L caused an accumulation of cells in the S phase and a decrease in the G0-G1 phase compared with control siRNA-transfected cells(Figure 5C, p < 0.05). Cells transfected with MACC1-siRNA1 and MACC1-siRNA2 showed significantly increased apoptotic rates as compared to control siRNA-transfected cells (MHCC 97H: 20.1%, 16.9% vs 6.95%, p < 0.05; MHCC 97L: 17.7%, 15.4% vs 5.46%, p < 0.05) (Figure 5D). Above results indicate that expression of MACC1 are associated with tumor cell proliferation and apoptosis.

## Discussion

It is well known that the patients with advanced HCC carry extremely poor outcomes [27]. These patients generally do not tolerate, nor response to systemic chemotherapy and local radiation therapy [28]. Thus, the treatment options for these patients remain limited. Although sorafenib may improve outcomes in these patients [5,6], the patient tolerance is usually low, the effects remain limited, and the cost could be high for this option. Thus, developing new therapies to this group of HCC patients is urgently needed. Cryoablation has been shown to induce growth inhibition of unresectable tumor and offers several potent advantages versus other ablation methods [29], but the data on cryoablation for advanced HCC remains very limited. Wilson et al. reported that cryoablation was a promising therapy for advanced HCC with potential benefits in prolonging patient survival [8]*.* A recent cohort study indicated that cryotherapy is safe and effective for unresectable HCC or recurrent HCC [12].

In the present study, we prospectively analyzed 120 cases with BCLC stage C unresectable HCC, underwent cryoablation, the largest sample size in this type of study to our best knowledge. According to the historical studies with the compared of patient populations, despite RFA provided the median OS of 8.5 months and TTP of 4.2 months in this type of patients, the reported after RFA for unresectable advanced HCC have not got any a case of CR [30]. Our data showed that cryoablation in patients with BCLC stage C unresectable HCC resulted in a significantly improved median post cryoablation OS (10.5 months) and TTP (5.5 months) with CER and DCR being 16.7% and 62.5%, respectively. Especially, five (4.2%) of these patients showed growth inhibition of non-treated tumor induced by post-cryoablation and 3 of them be alive up to the end of the follow-up (Figure 1). Thus, our findings further indicated that besides HCC ablation, cryotherapy might also function as a systemic treatment by improved immunity, indicated a comparable or even better OS and TTP, and other survival segregates of cryoablation in patients with advanced stage of HCC, compared to other current standard therapies, such as percutaneous ethanol injection and RFA as historically reported [31,32]. In addition, cryoablation has several advantages as follow. First, the cryoablation has the ability to produce larger and more precise zones of ablation [33]. Second, the frozen tissue is identified as a hyperechoic boundary with dense posterior shadowing, which allows excellent visualization of the nearest aspect of the ablation zone can be carefully monitored by US or CT or MRI [34,35]. Third, percutaneous cryoablation produce mild related-pain without general anaesthesia [36]. Last, tumour seeding after percutaneous cryoablation for HCC is low [37-39]. Our data support further randomized multicenter clinical trials to validate our findings.

Previous studies showed cryoablation was associated with 11% major complications [40,41]. We found although the majority was minor complications, severe complications, such as hepatorrhexis bleeding and Cryoshock syndrome, occured in 6.7% patients. To our experience [24], tumors with larger size, subcapsule location without encompassed liver parenchyma or adjacent to the gallbladder or loops of bowel will increase the risk of severe complications. Inserting the cryoprobe across a portion of normal hepatic parenchyma for subcapsular tumours can in some degree minimise both liver haemorrhage and needle-tract seeding. Cryoablation could effectively spare the normal livers, but severe liver damage occurred occasionally in patients with compromised liver function (Child-Pugh classification score >8) or after a large area of ablation. We believe compromised liver function and total estimated area (TEA) should be considered to deliver the effective ablation of the tumors and avoid severe complications in patients with advanced HCC. In this corhort of patients, the 30-day post-cryoablation mortality rate was 0%, suggesting that cryoablation significantly improved clinical outcomes in these patients with acceptable tolerance and safety profiles as previously reported [8,40,41].

Our findings provide a strong rationale for not only further multicenter prospective studies to validate our results, but also studies on combination therapy of cryoablation with sorafenib. Indeed, our early single center study did support the feasibility of this combination therapy in HCC patients [42].

Although we demonstrated significant short-term therapeutic benefits of cryoablation, 76.7% patients died during post-cryoablation follow up. This is not surprising as these patients had Child class A-B cirrhosis, advanced HCC and 40.8% had imaging report of the main PVT. Most common etiology of mortality was variceal bleeding, likely due to severe portal hypertension secondary to portal vein thrombosis. Majority (63%) died of cirrhosis related complications and 37% died of HCC related complication, as previously reported [43].

MACC1 is a recently identified molecule involved in metastasis of colon cancers [14]. Previous studies on the association of MACC1 with HCC were largely focused on BCLC stage A, but not stage C HCC [17,18]. The present study included a uniform group of BCLC stage C HCC, providing a great opportunity to assess this issue. We found that those with a lower intratumoral MACC1 mRNA expression had significant higher CER (p = 0.027) and CDR (p = 0.023) than those with a higher intratumoral MACC1 mRNA expression. Consistent with these, those with a lower intratumoral MACC1 mRNA expression showed significantly longer median post cryoablation TTP and OS than those with a higher intratumoral mRNA MACC1 expression. Because overexpression of MACC1 induces down-stream activation of HGF/c-Met and promotes tumor cell growth as well as tumor cell invasion [15], clinically we found a higher intratumoral MACC1 mRNA level was significantly more associated with younger age, portal vessel trunk invasion, tumor size, tumor number and poorly tumor differentiation. Our data extended the previous findings [17,18] and suggested that intratumoral MACC1 mRNA expression might serve as a clinical surrogate for clinical presentation and post cryoablation outcomes in patients with HBV-related cirrhosis and BCLC stage C HCC.

Besides MACC1 mRNA expression, we also found that nuclear MACC1 protein overexpression was presented in 44.8% of tumorous samples from these patients with advanced HCC. Furthermore, nuclear MACC1 expression at the time of cryoablation was not only strongly associated with larger tumor size and poorly differentiation, but also associated with poor outcome in these patients. Those with nuclear MACC1 staining had shorter median TTP and OS than those with MACC1 cytoplasmic staining. These findings are consistent with that reported in primary colon cancers [14], and our recent report that MACC1 mRNA overexpression is associated with enhanced tumor progression and may serve as a surrogate to predict recurrence or metastasis after hepatectomy [17]. Although the precise underlying mechanism remains to be determined, in vitro we found that the HCC cell lines with highly metastasis potential, especially MHCC-97H and MHCC-97 L [44], had a relatively high MACC1 and c-Met expression, whereas suppressing of MACC1 by si-RNA significantly reduced c-Met expression. Our data further confirmed that silencing of MACC1 resulted in c-Met suppressed expression, cell cycle arrest and apoptosis induction. These results shed new insights into the molecular mechanisms involved in the progression and prognosis of HCC, it seems that the nuclear translocation of MACC1 may be associated with c-Met and co-contribute to the pathogenesis of HCC [18,45] and affect outcomes of advanced HCC. Distribution of MACC1 expression in HCC cells may help us to select the best appropriate patients for cryotherapy, since the absence of therapeutic alternatives for advanced HCC.

Multivariable analysis showed that high intratumoral MACC1 mRNA expression, together with ECOG PS, predict the outcomes of post-cryoablation treatment in patients with unresectable and BCLC stage C HCC. It should be noted that besides MACC1 overexpression, we also found that ECOG PS was independently associated with OS, which was consistent with the results of previous studies [46]. Ideally, the tumor control rate increases with the completion of local treatment. Patients with a better ECOG PS had the opportunity for successful local treatment because of acceptable adverse effects. However, some well-established prognostic predictors, such as tumor differentiation, HCC tumor size and alpha-fetoprotein level were not found to be associated with post cryoablation outcomes in the present study. PVT is generally accepted to be the most independent factors affecting survival in HCC patients [47], but the sub-location of thrombus, whether in branch or trunk portal vein, was also excluded from multivariable analysis. Because the HCC patients in previous studies included different staging, the baseline PVT was a significant predictor for worse survival in HCC patients [48]. Considering that all of patients have PVT in current study, it was as one of the inclusion criteria and an identical factor that may contribute to the prognosis between patients with high or low MACC1 expression. Hence, PVT was excluded from multivariate analysis.

## Conclusions

The present study demonstrated that cryoablation is a safe and effective therapy for BCLC stage C unresectable HCC with Child class A or B. MACC1 mRNA and protein nuclear overexpression was associated with clinical presentation of more advanced HCC and poor outcomes after cryoablation. It should be noted, this was an uncontrolled, prospective followed study, further randomized and control studies are needed to validate our findings.

## Abbreviations

MACC1: Metastasis-associated in colon cancer-1; HCC: Hepatocellular carcinoma; TTP: Time to progression; OS: Overall survival; RFA: Radiofrequency Ablation; TAE: Transcatheter arterial embolization; CER: Clinical efficacy response; DCR: Disease-control rate

## Competing interests

The authors declare that they have no competing interests (Additional file 1).

## Authors’ contributions

YPY, JHQ and KQH conceived and designed the experiments. XJC and ZXX Performed the experiments. YPY, JHQ and XJC analyzed the data. WLB, YYL, ZD, HW, LJA, CPW and ZZ contributed reagents, materials or analysis tools. YPY and KQH wrote the paper. All authors read and approved the final manuscript.

## Supplementary Material

Additional file 1 BioMed Central copyright and license agreement. Click here for file
