# Supplementary material for: High intratumoral metastasis-associated in colon cancer-1 expression predicts poor outcomes of cryoablation therapy for advanced hepatocellular carcinoma
Source: J Transl Med. 2013 Feb 15;11:41. doi: 10.1186/1479-5876-11-41 (PMC3599141; doi:10.1186/1479-5876-11-41)
Supplement: Additional file 1 — BioMed Central copyright and license agreement. [file 1479-5876-11-41-S1.pdf]

BioMed Central copyright and license agreement

In submitting a research article ('article') to any of the journals published by BioMed Central Ltd ('BioMed Central') I certify that:

1. I am authorized by my co-authors to enter into these arrangements.

2. I warrant, on behalf of myself and my co-authors, that:

   a. the article is original, has not been formally published in any other peer-reviewed journal, is not under consideration by any other journal and does not infringe any existing copyright or any other third party rights;

   b. I am/we are the sole author(s) of the article and have full authority to enter into this agreement and in granting rights to BioMed Central are not in breach of any other obligation. If the law requires that the article be published in the public domain, I/we will notify BioMed Central at the time of submission upon which clauses 3 through 6 inclusive do not apply;

   c. the article contains nothing that is unlawful, libellous, or which would, if published, constitute a breach of contract or of confidence or of commitment given to secrecy;

   d. I/we have taken due care to ensure the integrity of the article. To my/our - and currently accepted scientific - knowledge all statements contained in it purporting to be facts are true and any formula or instruction contained in the article will not, if followed accurately, cause any injury, illness or damage to the user.

And I agree to the following license agreement:

BioMed Central Open Access license agreement

Brief summary of the agreement

Anyone is free:

- to copy, distribute, and display the work;
- to make derivative works;
- to make commercial use of the work;

Under the following conditions: Attribution

- the original author must be given credit;
- for any reuse or distribution, it must be made clear to others what the license terms of this work are;
- any of these conditions can be waived if the authors gives permission.

Statutory fair use and other rights are in no way affected by the above.

Full BioMed Central Open Access license agreement

(Identical to the 'Creative Commons Attribution License')

License

THE WORK (AS DEFINED BELOW) IS PROVIDED UNDER THE TERMS OF THIS BIOMED CENTRAL OPEN ACCESS LICENSE ("LICENSE"). THE WORK IS PROTECTED BY COPYRIGHT AND/OR OTHER APPLICABLE LAW. ANY USE OF THE WORK OTHER THAN AS AUTHORIZED UNDER THIS LICENSE IS PROHIBITED.

BY EXERCISING ANY RIGHTS TO THE WORK PROVIDED HERE, YOU ACCEPT AND AGREE TO BE BOUND BY THE TERMS OF THIS LICENSE. THE LICENSOR GRANTS YOU THE RIGHTS CONTAINED HERE IN CONSIDERATION OF YOUR ACCEPTANCE OF SUCH TERMS AND CONDITIONS.

1. Definitions

a. **"Collective Work"** means a work, such as a periodical issue, anthology or encyclopedia, in which the Work in its entirety in unmodified form, along with a number of other contributions, constituting separate and independent works in themselves, are assembled into a collective whole. A work that constitutes a Collective Work will not be considered a Derivative Work (as defined below) for the purposes of this License.

b. **"Derivative Work"** means a work based upon the Work or upon the Work and other pre-existing works, such as a translation, musical arrangement, dramatization, fictionalization, motion picture version, sound recording, art reproduction, abridgment, condensation, or any other form in which the Work may be recast, transformed, or adapted, except that a work that constitutes a Collective Work will not be considered a Derivative Work for the purpose of this License. For the avoidance of doubt, where the Work is a musical composition or sound recording, the synchronization of the Work in timed-relation with a moving image ("synching") will be considered a Derivative Work for the purpose of this License.

c. **"Licensor"** means the individual or entity that offers the Work under the terms of this License.

d. **"Original Author"** means the individual or entity who created the Work.

e. **"Work"** means the copyrightable work of authorship offered under the terms of this License.

f. **"You"** means an individual or entity exercising rights under this License who has not previously violated the terms of this License with respect to the Work, or who has received express permission from the Licensor to exercise rights under this License despite a previous violation.

2. Fair Use Rights
Nothing in this license is intended to reduce, limit, or restrict any rights arising from fair use, first sale or other limitations on the exclusive rights of the copyright owner under copyright law or other applicable laws.

3. License Grant
Subject to the terms and conditions of this License, Licensor hereby grants You a worldwide, royalty-free, non-exclusive, perpetual (for the duration of the applicable copyright) license to exercise the rights in the Work as stated below:

a. to reproduce the Work, to incorporate the Work into one or more Collective Works, and to reproduce the Work as incorporated in the Collective Works;

b. to create and reproduce Derivative Works;

c. to distribute copies or phonorecords of, display publicly, perform publicly, and perform publicly by means of a digital audio transmission the Work including as incorporated in Collective Works;

d. to distribute copies or phonorecords of, display publicly, perform publicly, and perform publicly by means of a digital audio transmission Derivative Works;

e. For the avoidance of doubt, where the work is a musical composition:

   i. **Performance Royalties Under Blanket Licenses.** Licensor waives the exclusive right to collect, whether individually or via a performance rights society (e.g. ASCAP, BMI, SESAC), royalties for the public performance or public digital performance (e.g. webcast) of the Work.

   ii. **Mechanical Rights and Statutory Royalties.** Licensor waives the exclusive right to collect, whether individually or via a music rights agency or designated agent (e.g. Harry Fox Agency), royalties for any phonorecord You create from the Work ("cover version") and distribute, subject to the compulsory license created by 17 USC Section 115 of the US Copyright Act (or the equivalent in other jurisdictions).

f. **Webcasting Rights and Statutory Royalties.** For the avoidance of doubt, where the Work is a sound recording, Licensor waives the exclusive right to collect, whether individually or via a performance-rights society (e.g. SoundExchange), royalties for the public digital performance (e.g. webcast) of the Work, subject to the compulsory license created by 17 USC Section 114 of the US Copyright Act (or the equivalent in other jurisdictions).

The above rights may be exercised in all media and formats whether now known or hereafter devised. The above rights include the right to make such modifications as are technically necessary to exercise the rights in other media and formats. All rights not expressly granted by Licensor are hereby reserved.

4. Restrictions
The license granted in Section 3 above is expressly made subject to and limited by the following restrictions:

a. You may distribute, publicly display, publicly perform, or publicly digitally perform the Work only under the terms of this License, and You must include a copy of, or the Uniform Resource Identifier for, this License with every copy or phonorecord of the Work You distribute, publicly display, publicly perform, or publicly digitally perform. You may not offer or impose any terms on the Work that alter or restrict the terms of this License or the recipients' exercise of the rights granted hereunder. You may not sublicense the Work. You must keep intact all notices that refer to this License and to the disclaimer of warranties. You may not distribute, publicly display, publicly perform, or publicly digitally perform the Work with any technological measures that control access or use of the Work in a manner inconsistent with the terms of this License Agreement. The above applies to the Work as incorporated in a Collective Work, but this does not require the Collective Work apart from the Work itself to be made subject to the terms of this License. If You create a Collective Work, upon notice from any Licensor You must, to the extent practicable, remove from the Collective Work any reference to such Licensor or the Original Author, as requested. If You create a Derivative Work, upon notice from any Licensor You must, to the extent practicable, remove from the Derivative Work any reference to such Licensor or the Original Author, as requested.

b. If you distribute, publicly display, publicly perform, or publicly digitally perform the Work or any Derivative Works or Collective Works, You must keep intact all copyright notices for the Work and give the Original Author credit reasonable to the medium or means You are utilizing by conveying the name (or pseudonym if applicable) of the Original Author if supplied; the title of the Work if supplied; to the extent reasonably practicable, the Uniform Resource Identifier, if any, that Licensor specifies to be associated with the Work, unless such URI does not refer to the copyright notice or licensing information for the Work; and in the case of a Derivative Work, a credit

identifying the use of the Work in the Derivative Work (e.g., "French translation of the Work by Original Author," or "Screenplay based on original Work by Original Author"). Such credit may be implemented in any reasonable manner; provided, however, that in the case of a Derivative Work or Collective Work, at a minimum such credit will appear where any other comparable authorship credit appears and in a manner at least as prominent as such other comparable authorship credit.

5. Representations, Warranties and Disclaimer

UNLESS OTHERWISE MUTUALLY AGREED TO BY THE PARTIES IN WRITING, LICENSOR OFFERS THE WORK AS-IS AND MAKES NO REPRESENTATIONS OR WARRANTIES OF ANY KIND CONCERNING THE WORK, EXPRESS, IMPLIED, STATUTORY OR OTHERWISE, INCLUDING, WITHOUT LIMITATION, WARRANTIES OF TITLE, MERCHANTIBILITY, FITNESS FOR A PARTICULAR PURPOSE, NONINFRINGEMENT, OR THE ABSENCE OF LATENT OR OTHER DEFECTS, ACCURACY, OR THE PRESENCE OF ABSENCE OF ERRORS, WHETHER OR NOT DISCOVERABLE. SOME JURISDICTIONS DO NOT ALLOW THE EXCLUSION OF IMPLIED WARRANTIES, SO SUCH EXCLUSION MAY NOT APPLY TO YOU.

6. Limitation on Liability

EXCEPT TO THE EXTENT REQUIRED BY APPLICABLE LAW, IN NO EVENT WILL LICENSOR BE LIABLE TO YOU ON ANY LEGAL THEORY FOR ANY SPECIAL, INCIDENTAL, CONSEQUENTIAL, PUNITIVE OR EXEMPLARY DAMAGES ARISING OUT OF THIS LICENSE OR THE USE OF THE WORK, EVEN IF LICENSOR HAS BEEN ADVISED OF THE POSSIBILITY OF SUCH DAMAGES.

7. Termination

a. This License and the rights granted hereunder will terminate automatically upon any breach by You of the terms of this License. Individuals or entities who have received Derivative Works or Collective Works from You under this License, however, will not have their licenses terminated provided such individuals or entities remain in full compliance with those licenses. Sections 1, 2, 5, 6, 7, and 8 will survive any termination of this License.

b. Subject to the above terms and conditions, the license granted here is perpetual (for the duration of the applicable copyright in the Work). Notwithstanding the above, Licensor reserves the right to release the Work under different license terms or to stop distributing the Work at any time; provided, however that any such election will not serve to withdraw this License (or any other license that has been, or is required to be, granted under the terms of this License), and this License will continue in full force and effect unless terminated as stated above.

8. Miscellaneous

a. Each time You distribute or publicly digitally perform the Work or a Collective Work, the Licensor offers to the recipient a license to the Work on the same terms and conditions as the license granted to You under this License.

b. Each time You distribute or publicly digitally perform a Derivative Work, Licensor offers to the recipient a license to the original Work on the same terms and conditions as the license granted to You under this License.

c. If any provision of this License is invalid or unenforceable under applicable law, it shall not affect the validity or enforceability of the remainder of the terms of this License, and without further action by the parties to this agreement, such provision shall be reformed to the minimum extent necessary to make such provision valid and enforceable.

d. No term or provision of this License shall be deemed waived and no breach consented to unless such waiver or consent shall be in writing and signed by the party to be charged with such waiver or consent.

e. This License constitutes the entire agreement between the parties with respect to the Work licensed here. There are no understandings, agreements or representations with respect to the Work not specified here. Licensor shall not be bound by any additional provisions that may appear in any communication from You. This License may not be modified without the mutual written agreement of the Licensor and You.
